# Diagnose und Therapie der Granulomatose mit Polyangiitis und mikroskopische Polyangiitis – 2023: Konsens-Empfehlungen der Österreichischen Gesellschaften für Nephrologie (ÖGN) & Rheumatologie (ÖGR)

**DOI:** 10.1007/s00508-023-02262-9

**Published:** 2023-09-20

**Authors:** Balazs Odler, Martin Windpessl, Kathrin Eller, Marcus D. Säemann, Karl Lhotta, Irmgard Neumann, Gregor Öberseder, Christina Duftner, Christian Dejaco, Michael Rudnicki, Philipp Gauckler, Rainer Hintenberger, Jochen Zwerina, Jens Thiel, Andreas Kronbichler

**Affiliations:** 1 Klinische Abteilung für Nephrologie, Abteilung für Innere Medizin III (Nephrologie, Dialyse und Hypertensiologie), Medizinische Universität Graz, Graz, Österreich; 2 Abteilung für Innere Medizin IV, Klinikum Wels-Grieskirchen, Wels, Österreich; 3 Medizinische Fakultät, JKU, Linz, Österreich; 4 6. Medizinische Abteilung mit Nephrologie & Dialyse, Klinik Ottakring, Wien, Österreich; 5 Medizinische Fakultät, SFU, Wien, Österreich; 6 Abteilung für Innere Medizin III (Nephrologie, Dialyse und Hypertensiologie), Akademisches Lehrkrankenhaus Feldkirch, Feldkirch, Österreich; 7 Vasculitis.at, Wien, Österreich; 8 Immunologiezentrum Zürich (IZZ), Zürich, Schweiz; 9 Innere Medizin, Wien, Österreich; 10 Department Innere Medizin II, Medizinische Universität Innsbruck, Innsbruck, Österreich; 11 Rheumatologie, Krankenhaus Bruneck, Bruneck, Italien; 12 Department Innere Medizin IV (Nephrologie und Hypertensiologie), Medizinische Universität Innsbruck, Innsbruck, Österreich; 13 Abteilung Innere Medizin 2 (Gastroenterologie und Hepatologie, Endokrinologie und Stoffwechsel, Nephrologie, Rheumatologie), JKU, Linz, Österreich; 14 1. Medizinische Abteilung, Hanusch Krankenhaus, Wien, Österreich; 15 Klinische Abteilung für Rheumatologie und Immunologie, Bereich Innere Medizin, Medizinische Universität Graz, Graz, Österreich

**Keywords:** ANCA-assoziierte Vaskulitiden (AAV), Rituximab, Avacopan, Diagnostik, Therapie, ANCA-associated vasculitides (AAV), Rituximab, Avacopan, Diagnostics, Therapy

## Abstract

ANCA-assoziierte Vaskulitiden (AAV) sind seltene, komplexe systemische Erkrankungen, die aufgrund unspezifischer klinischer Symptome zum Zeitpunkt der Konsultation oft schwer zu diagnostizieren sind. Der klinische Verlauf kann jedoch sehr schwerwiegend und sogar lebensbedrohlich sein und eine sofortige Diagnose und Behandlung erfordern.

Daher ist es wichtig, die Ärzteschaft für diese Erkrankung zu sensibilisieren und Kolleg*innen zu unterstützen, die nicht regelmäßig mit diesen seltenen Krankheiten konfrontiert sind. Die Österreichische Gesellschaft für Nephrologie (ÖGN) und die Österreichische Gesellschaft für Rheumatologie (ÖGR) stellen hier einen gemeinsamen Konsens darüber vor, wie Patient*innen mit Granulomatose mit Polyangiitis (GPA) und mikroskopischer Polyangiitis (MPA) am besten diagnostiziert und behandelt werden können.

## Abstract (en)

ANCA-associated vasculitides (AAV) are rare, complex systemic diseases that are often difficult to diagnose, because of unspecific clinical symptoms at presentation. However, the clinical course may be very dramatic and even life-threatening, necessitating prompt diagnosis and treatment.

Therefore, it is important to increase disease awareness among physicians and support colleagues who are not confronted with these rare diseases on a regular basis. Here, the Austrian Society of Nephrology (ÖGN) and the Austrian Society of Rheumatology (ÖGR) provide a joint consensus on how to best diagnose and manage patients with granulomatosis with polyangiitis (GPA) and microscopic polyangiitis (MPA).

## Einleitung

Anti-Neutrophile zytoplasmatische Antikörper (ANCA)-assoziierte Vaskulitiden (AAV) sind seltene Autoimmunerkrankungen (Inzidenz von 20–25 pro Millionen Menschen). Sie gliedern sich in drei Kategorien: Granulomatose mit Polyangiitis (GPA), mikroskopische Polyangiitis (MPA) und eosinophile Granulomatose mit Polyangiitis (EGPA). In dem vorliegenden Konsens liegt der Fokus auf GPA und MPA. Aktuelle bevölkerungsbezogene Studien berichteten sogar über höhere Inzidenzraten [1], vermutlich aufgrund der weit verbreiteten Verwendung von ANCA-Tests und der besseren Erkennung dieser Krankheiten. AAV können die meisten Organe befallen und sind potenziell lebensbedrohlich. Die Niere ist bei bis zu 70 bis 80 % der Patient*innen mit GPA und MPA beteiligt, was für die Prognose von entscheidender Bedeutung ist. Durch neue Behandlungen wurden die Mortalitätsraten bei Patient*innen reduziert, und Infektionen (oftmals begünstigt durch die Anwendung von Steroiden [GC]) tragen stärker als die aktive Vaskulitis zur frühen Mortalität bei, während im späteren Verlauf Herz‑/Kreislauf-Erkrankungen, Malignität und Infektionen die dominierenden Faktoren sind [2]. Zudem hängen Prognose und Mortalität von den vaskulitisbedingten Schädigungen ab, und die Therapielast ist mit höheren Gesundheitsausgaben verbunden [3]. Frühzeitige Diagnose und Behandlung verbessern den Ausgang der Erkrankung; die verzögerte Diagnostik (Monate bis Jahre, im Durchschnitt etwa 3 Monate) [4] ist jedoch nach wie vor ein Dilemma. Deshalb ist es wichtig, alle am Krankheitsmanagement beteiligten Ärzt*innen für diese Erkrankung zu sensibilisieren. Da bei den meisten Patient*innen mehrere Organe betroffen sind, ist eine enge Zusammenarbeit zwischen den behandelnden Fachbereichen notwendig. In der Regel gilt dies für Hals‑, Nasen‑, Ohren-Ärzt*innen (HNO), Lungenärzt*innen, Rheumatolog*innen, Nephrolog*innen, Immunolog*innen, Hautärzt*innen und andere Ärzt*innen bei seltenerer Symptomatik (wie Beteiligung des Magen-Darm-Traktes oder der Prostata). Die Ansätze zur Diagnose von GPA und MPA werden in Tab. 1 dargestellt. Differenzialdiagnosen sind zu beachten, und sekundäre Formen im Rahmen von Infektionen, assoziiert mit Medikamenteneinnahme und im Rahmen anderer Erkrankungen mit positivem ANCA-Test (Tab. 2) müssen ausgeschlossen werden. Zusätzlich ist die Beteiligung von Patholog*innen wichtig, um eine AAV-Diagnose zu stellen, insbesondere in Fällen mit unsicherer Diagnose. Patholog*innen mit Fachkenntnis im Bereich Nierenerkrankungen werden benötigt, um Nierenbiopsien gemäß der Berden-Klassifikation und dem Brix-Score (ANCA Renal Risk Score (ARRS)) [5–7], international anerkannten Scores zur Vorhersage der potenziellen Wiederherstellung der Nierenfunktion und langfristigen Nierenüberlebensraten, auszuwerten. Das wichtigste Prognosemerkmal bei der Histologie der Nierenbiopsie ist nicht die Art und das Ausmaß glomerulonephritischer Läsionen, sondern der Anteil völlig intakter Glomeruli.

**Table 1:** 

Schritte	Spezifische Erwägungen
Symptome	Ein systemischer Ansatz ist geboten; die Organbeteiligung kann anhand des Birmingham Vasculitis Activity Scores beurteilt werden (online verfügbar unter: www.bvasvdi.org); es gilt zu beachten, dass andere Organmanifestationen wie Prostatitis oder Parotitis nicht erfasst werden und bei einem Teil der Patient*innen, insbesondere bei PR3-ANCA-Vaskulitis, vorliegen
Laborwerte („Immunologie“)	ANCA-Immunfluoreszenz, PR3- und MPO-Elisa (Line- oder Dot-Immunoassay), Anti-GBM-Antikörper, ANA, Rheumafaktor, Kryoglobuline, Antiphospholipid-Antikörper, C3/C4, C1q-Antikörper, dsDNA Immunglobuline (zunächst IgG, IgA, IgM; Hinweis: Niedrige IgG-Ausgangswerte lassen eine schwere Hypogammaglobulinämie nach der Behandlung vermuten)
Laborwerte („Urin“)	Erythrozyten, einschließlich Harnsediment Proteinurie (UPCR), Albuminurie (UACR)
Laborwerte („unspezifisch“)	CRP und BSG Großes Blutbild (Thrombozytose) PCT (sollte negativ oder nur leicht erhöht sein; bei positivem Befund sollte eine gleichzeitige aktive Infektion in Betracht gezogen werden) Hepatitis-Screening, HIV-Screening
Bildgebung und funktionelle Untersuchungen	HR-CT des Thorax Lungenfunktionstest (einschließlich DLCO) MR/CT der Nasennebenhöhlen bei entsprechender Symptomatik MR von verdächtigen Läsionen (Beteiligung der Ohrspeicheldrüse) Ultraschall des Abdomens Echokardiographie
Biopsie	Angezeigt bei unklarer Diagnostik Nierenbiopsie bei Hämaturie ± Proteinurie oder eingeschränkter Nierenfunktion Hinweis: Andere Zielorganbiopsien sind oft unspezifisch und müssen von erfahrenem medizinischem Personal durchgeführt werden (d. h. Biopsie des Nasengewebes ergibt oft unspezifische nekrotische Schleimhaut oder Probennahmefehler) Ein negatives Biopsieergebnis schließt die Diagnose einer ANCA-assoziierten Vaskulitis nicht aus
Überweisung	Grundsätzlich empfohlen; im Falle einer Unsicherheit ist eine Überweisung an ein Fachzentrum angezeigt

*ANA* Anti-nukleäre Antikörper, *ANCA* Anti-Neutrophile zytoplasmatische Antikörper, *CRP* C-reaktives Protein, *CT* Computertomographie, *dsDNA* Doppelstrang-DNA, *GBM* glomeruläre Basalmembran, *HR* high resolution, *MPO* Myeloperoxidase, *MR* Magnetresonanz, *PCT* Procalcitonin, *PR3* Proteinase 3

**Table 2:** 

Infektionen	*Staphylococcus aureus, Escherichia coli, Streptococcus viridans, Bartonella quintana, Bartonella henselae, Coxiella burnetii, Leishmania braziliensis, Rhizopus arrhizus, Nocardia asteroides, Pneumocystis jirovecii, Ehrlichia chaffeensis, Troponema pallidum. Plasmodium falciparum, Plasmodium vivax, Entamoeba histolytica* usw.
Wirkstoff-induziert	Hydralazin^a^, Prophylthiouracil^a^, Levamisol^a^, Phenytoin, Cefotaxim^a^, D‑Penicillamin, alpha-Methyldopa, Adalimumab, Golimumab, Atorvastatin, Simvastatin/Ezetimib, Interferon-alpha, Allopurinol, Sulfasalazin, Pantoprazol, mRNA-Impfstoffe, virale Vektorimpfstoffe usw
Diagnosen, die möglicherweise mit einem positiven ANCA-Test assoziiert sind	Entzündliche Darmerkrankung, Autoimmunhepatitis, systemischer Lupus erythematodes, Sklerodermie, Malignität, rheumatoide Arthritis, Sjögren-Syndrom, Antiphospholipid-Syndrom

^a^Substanzen mit einem wahrscheinlichen Zusammenhang zwischen Verwendung und Diagnose einer ANCA-assoziierten Vaskulitis. Die ANCA-Titer sind in sekundären Fällen generell niedriger, und die Tests bei diesen Patient*innen sind häufig „doppelt ANCA-positiv“, d. h. positiv auf Proteinase 3‑ und auf Myeloperoxidase-ANCA oder sonstige ANCA-Spezialformen. Die meisten dieser Patient*innen benötigen keine Erhaltungstherapie, nachdem die „Auslösersubstanz“ abgesetzt wird. Levamisol ist in Kokain-Präparaten enthalten. Deshalb ist die Häufigkeit von Levamisol-induzierter Vaskulitis in Gruppen mit starkem Kokainkonsum besonders hoch

Der erste gemeinsame Konsens der Österreichischen Gesellschaft für Nephrologie und Rheumatologie (ÖGN/ÖGR) zielt darauf ab, diagnostische und therapeutische Ansätze zur Behandlung von AAV-Patient*innen zusammenzufassen. Er behandelt jedoch auch den bisher nicht erfüllten Bedarf in Österreich, beispielsweise im Hinblick auf die Implementierung eines mit der paneuropäischen Datenbank verlinkten Registers und Hilfsorganisationen für Patient*innen wie die Vasculitis UK (gegründet in 2009).

## Wie wird die Diagnose einer ANCA-assoziierten Vaskulitis gestellt?

Patient*innen mit AAV haben in der Regel eine Vielzahl unspezifischer Symptome wie Unwohlsein (60 %), Fieber (35 %), Gelenkschmerzen (45 %) [8], was auf einen systemischen Entzündungsprozess hinweist, der oftmals dem Beginn der Erkrankung vorausgeht. Eine nicht unbedeutende Minderheit weist nicht die „klassischen“ Lungen- bzw. Nierensymptome auf, darunter besonders häufig neurologische (Mononeuritis multiplex) oder dermale Symptomatiken (leukozytoklastische Vaskulitis). HNO-Symptome sind häufig bei PR3-ANCA-assoziierten AAV (> 70 %) festzustellen [9]. Daher konsultieren viele Patient*innen verschiedene Ärzt*innen, bevor eine Diagnose gestellt wird, und werden aufgrund erhöhter Entzündungsparameter mit verschiedenen Antibiotika behandelt. Der CRP-Spiegel (C-reaktives Protein; in unterschiedlichem Ausmaß) und die Blutsenkungsgeschwindigkeit (BSG, meist über 50 mm nach 1 h) sind bei neu diagnostizierten Patient*innen erhöht [10], während die Bestimmung von Procalcitonin (PCT) in der Regel negative oder nur leicht erhöhte Ergebnisse zeigt, wenn keine aktiven Infektionen vorliegen [11, 12]. Dennoch schließt ein negativer/leicht erhöhter PCT-Wert das Vorliegen einer aktiven Vaskulitis nicht aus, insbesondere wenn die Symptome auf das Vorliegen einer AAV hindeuten. Bei Patient*innen mit konstitutionellen Symptomen und erhöhten Entzündungsparametern, die nicht auf eine Antibiotikatherapie ansprechen, ist es wichtig, AAV als Differentialdiagnose in Betracht zu ziehen.

Bei Patient*innen mit Verdacht auf GPA oder MPA empfehlen wir, den in Tab. 1 dargelegten diagnostischen Ansatz zu befolgen. Ferner empfehlen wir eine systematische Vorgehensweise zur Bewertung der Krankheitsaktivität, beispielsweise die Verwendung des Birmingham Vasculitis Activity Scores (BVAS, online verfügbar unter www.bvasvdi.org). Von Patient*innen angegebene Symptome, die im BVAS nicht erfasst werden, sollten systematisch beurteilt werden, und es können zusätzliche diagnostische Schritte erforderlich sein, z. B. Messungen des prostataspezifischen Antigens bei Verdacht auf Prostatitis oder Ultraschall als erste bildgebende Methode im Falle einer Skelettbeteiligung oder Parotitis. Die Patient*innen sollten einer Ultraschalluntersuchung des Abdomens, einem Lungenfunktionstest (einschließlich Messung der Diffusionskapazität der Lunge für Kohlenmonoxid (DLCO) bei Verdacht auf eine interstitielle Lungenerkrankung) und einer hochauflösenden Computertomographie des Thorax unterzogen werden, um eine Lungenbeteiligung auszuschließen oder zu bestätigen (z. B. Granulome bei PR3-ANCA-Vaskulitis oder interstitielle Lungenerkrankung (ILD) bei einer MPO-ANCA-Vaskulitis). Es kann zudem eine alveoläre Hämorrhagie erkannt werden, bevor Symptome (Hämoptyse) einsetzen. Insbesondere eine starke alveoläre Hämorrhagie ist mit einer schlechten Prognose assoziiert und erfordert eine schnelle und intensivere Behandlung (siehe weiter unten) [13].

Weitere Laboruntersuchungen wie ANCA-Tests mittels Immunfluoreszenz und die Bestimmung von PR3- und MPO-ANCA-Autoantikörpern mittels ELISA sollten ebenfalls durchgeführt werden. Es können auch Line- oder Dot-Immunoassays durchgeführt werden. Glomeruläre Basalmembranantikörper (GBM) sind bei einem signifikanten Anteil der Patient*innen positiv (bei etwa 5 % liegt eine doppelt positive Erkrankung vor) [14]. Auch eine Bestimmung der Komplementkomponente C3 (mit einer schlechteren Prognose und Nierenbeteiligung assoziiert), [8, 15], Urinanalyse (Vorliegen von Erythrozytenzylindern und Proteinurie) sowie organspezifische Untersuchungen (wie Serumkreatinin, Leber- und Lungenfunktionstests, usw.) sind sinnvoll (Tab. 1). In Fällen mit diagnostischer Unsicherheit kann eine Biopsie der betroffenen Organe angezeigt sein. Die Quote „negativer“ Biopsiebefunde an bestimmten Stellen (z. B. HNO-Trakt/Lunge < 50 %) ist hoch [16, 17] und könnte mit der Tatsache zusammenhängen, dass die Biopsie von unerfahrenen Ärzt*innen durchgeführt werden. Daher ist eine Überweisung an Spezialisten angezeigt, um die Häufigkeit falsch negativer Biopsien zu begrenzen. Darüber hinaus ist es sehr schwierig, primäre vaskulitische Läsionen von sekundären Gefäßentzündungen in einer stark entzündeten Gewebeumgebung zu unterscheiden. Diese Differenzierung ist bei Nierenbiopsien viel einfacher, da Glomeruli nicht von Entzündungen des umgebenden Gewebes befallen werden und glomerulonephritische Läsionen somit zuverlässige Indikatoren für Autoimmunschädigungen sind. Darüber hinaus ist das Muster der glomerulären Schädigung bei AAV (pauci-immun nekrotisierend/halbmondförmig) in den meisten Fällen sehr charakteristisch. Eine Nierenbiopsie ist angezeigt, wenn eine Nierenbeteiligung vermutet wird und insbesondere, wenn diagnostische Unsicherheiten bestehen; vor allem kann eine Hämaturie auf eine bedeutende Nierenpathologie hindeuten, selbst wenn die Nierenfunktion intakt ist [18]. Ein dringender Verdacht ergibt sich in der Regel bei der Feststellung von Erythrozytenzylindern im Urin („Urinsediment“) oder beim Vorliegen von Blut bei Analysen mit Urinteststreifen. Eine Nierenbiopsie hilft bei der Prognosestellung, insbesondere bei eingeschränkter Nierenfunktion, da mit ihr eine Vorhersage über eine mögliche Wiederherstellung der Nierenfunktion getroffen werden kann [5–7, 19]. Vor allem bei Fällen mit „begrenzter Erkrankung“, bei denen definitionsgemäß vorwiegend der HNO-Trakt und die Lungen betroffen sind, gibt es einen bestimmten Prozentsatz (etwa 10 %) an ANCA-negativen Befunden [20]. Hier sind weitere diagnostische Untersuchungen wie etwa ein positiver Biopsiebefund angezeigt, um die Diagnose einer GPA oder MPA zu untermauern. Vor der Einleitung einer Immunsuppression sind bestimmte Laborparameter notwendig, wie etwa das Ausschließen einer HIV-Infektion (Humanes Immundefizienz-Virus), von Hepatitis B/C (HBV/HBC) sowie die Bestimmung der Immunglobulin (Ig)-Konzentrationen.

Der ANCA Serotyp ist zudem hilfreich um die Aktivität der Erkrankung (nach Therapiebeginn) einzuschätzen. Neuere Untersuchungen haben gezeigt, dass es bei Patient*innen mit PR3-ANCA-Vaskulitis und schwerer Nierenbeteiligung nach Einleitung einer adäquaten immunsuppressiven Therapie zu einer weiteren Nierenfunktionsstörung kommen könnte, obwohl bei diesen Patient*innen die Erholungsrate der Nierenfunktion höher ist als bei MPO-ANCA Vaskulitis Patient*innen [21]. Ein spezifisches Verständnis dieser Kinetik ist wichtig, um die Intensität der Immunsuppression anzupassen und eine individuelle Prognose zu stellen. Es sollte beachtet werden, dass eine Verzögerung zwischen Diagnose und Einleiten der Therapie möglicherweise zu irreversiblen Organschäden führen könnte. Zudem liegt bei manchen GPA- oder MPA-Patient*innen (etwa 5 %) eine Beteiligung großer Gefäße vor, die an eine Vaskulitis der großen Gefäße erinnert; ein ANCA-Test kann hier in unklaren Fällen eine definitive Diagnose ermöglichen [22]. Die Diagnoseschritte und die Differentialdiagnose von AAV sind in Tab. 1 und 2 zusammengefasst.

## Der Nutzen eines seriellen ANCA-Screenings

Die Pathogenese der AAV ist komplex und umfasst Umwelt- und genetische Faktoren. Obwohl ANCA lediglich eine moderate Korrelation mit der Krankheitsaktivität aufweisen, werden in der klinischen Praxis serielle Bestimmungen der Myeloperoxidase- (MPO)- bzw. Proteinase-3-(PR3) -ANCA empfohlen. Dabei sind bestimmte Konstellationen beim Follow-up mit Rezidiven assoziiert: In einer holländischen Studie wurde ein Zusammenhang zwischen steigenden ANCA-Titern und Rezidiven der Erkrankung festgestellt, wobei dieser Zusammenhang bei Patient*innen mit Nierenbeteiligung stärker ausgeprägt war. Dies kann bei PR3-ANCA-Vaskulitis im Vergleich zu MPO-ANCA-Vaskulitis besonders häufig beobachtet werden [23]. Analog dazu sprach bei japanischen Patient*innen ein Anstieg des MPO-ANCA-Titers stark für Rezidive bei Patient*innen mit Nierenbeteiligung [24]. Die Induktions- und Erhaltungstherapie mit Rituximab (RTX) führt häufiger zu einer ANCA-Negativität, und die Aufrechterhaltung der Negativität ist mit einer länger anhaltenden Remission verbunden [25]. Bei Patient*innen, die eine Induktionstherapie mit Cyclophosphamid (CYC) erhielten, reduzierte die ANCA-Negativität zum Zeitpunkt der Umstellung auf die Erhaltungstherapie ebenfalls die Wahrscheinlichkeit eines Rezidivs [26]. Trotz dieser klaren Zusammenhänge besteht Konsens darüber, dass Behandlungsentscheidungen nicht allein auf der Grundlage von Veränderungen der ANCA-Titer getroffen werden sollten. Dies spiegelt sich auch in den Empfehlungen der European League of Associations for Rheumatology (EULAR) 2022 wider [27].

## Induktionstherapie bei GPA und MPA

Die Behandlung von GPA und MPA basiert im Allgemeinen auf der Schwere des klinischen Befundes, es sollte aber auch der zeitliche Verlauf der Symptomentwicklung berücksichtigt werden, um die Akuität der Erkrankung abzuschätzen. Für mehrere Behandlungsoptionen einer neu einsetzenden Vaskulitis liegt fundierte Evidenz vor, da für sie groß angelegte, randomisierte kontrollierte Studien (RCT) durchgeführt wurden. Das Behandlungsziel ist das Erreichen einer Remission. Diese wird in der Regel innerhalb der ersten 3 bis 6 Monate nach der Initialtherapie (Induktionstherapie) erreicht, wonach die Therapie umgestellt wird, um den Remissionszustand während des Follow-ups aufrecht zu erhalten.

**Figure 1:**
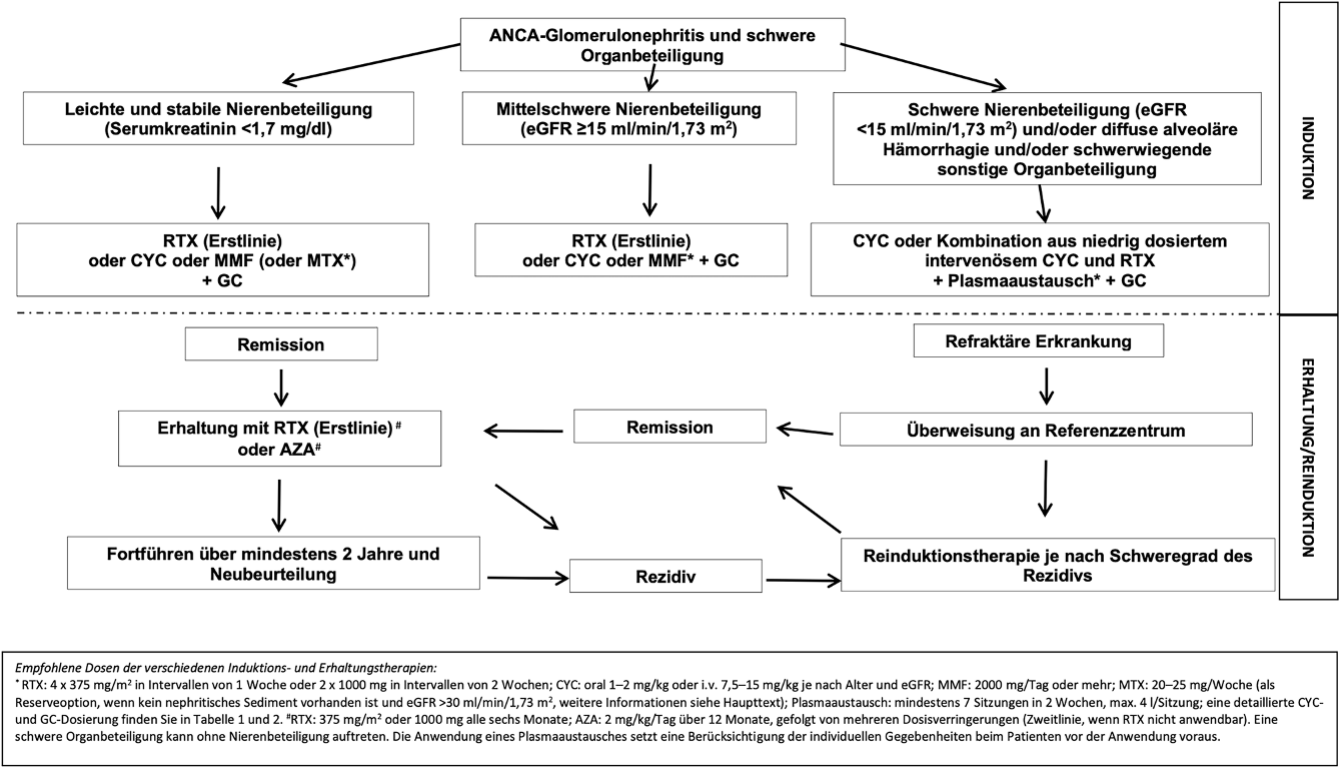

Die Behandlungsoptionen zur Remissionsinduktion hängen im Wesentlichen vom Schweregrad der Erkrankung ab (siehe Abb. 1). Bei Patient*innen mit Nierenbeteiligung und einer geschätzten glomerulären Filtrationsrate (eGFR) > 15 ml/min/1,73 m^2^ oder AAV ohne Nierenbeteiligung ist die Kombination von GC mit entweder einem RTX-Zyklus (4 Mal 375 mg/m^2^ oder 2 Mal 1000 mg) genauso wirksam wie orales (2 mg/kg Körpergewicht, mit Dosisanpassungen) oder intravenös verabreichtes CYC (15 mg/kg Körpergewicht, mit Dosisanpassungen – Tab. 3). Die intravenöse Gabe von CYC wird der oralen Verabreichung vorgezogen, da so die kumulierte Dosis reduziert werden kann, während die Remissionsraten identisch sind [28]. Bei stark ausgeprägten Formen (schwere alveoläre Hämorrhagie oder Nierenbeteiligung mit einer eGFR ≤ 15 ml/min/1,73 m^2^) liegt begrenzte Evidenz aus retrospektiven Studien sowie aus einer RCT (PEXIVAS-Studie) vor, die für eine RTX-Monotherapie spricht, wie in den KDIGO Leitlinien 2021 erläutert wird [29]. Ein RITUXVAS-Ansatz [30], bei dem intravenöses CYC mit RTX kombiniert wird, um eine Remission herbeizuführen, könnte in diesen Szenarien wirksamer sein als die Behandlung mit einem einzelnen Wirkstoff. Aktuelle Daten deuten darauf hin, dass ein solcher Ansatz ein schnelleres Ausschleichen von GC ermöglichen würde, ein entscheidender Faktor, um GC-Nebenwirkungen zu begrenzen (z. B. neu auftretender Diabetes mellitus [DM] und Verringerung der Knochenmineraldichte (BMD)). Daten aus der Praxis zeigen jedoch nach wie vor, dass eine langfristige GC-Anwendung (über 6 Monate hinaus) bei Patient*innen mit AAV häufig ist und etwa 75 % weiterhin GC erhalten [31], obwohl der Nutzen einer verlängerten GC-Verordnung, insbesondere bei RTX-induzierten Patient*innen, unklar bleibt. Ein überwiegender Großteil der Evidenz deutet darauf hin, dass eine Reduktion der kumulativen GC-Dosis die Zahl von schwerwiegenden Infektionen verringern kann, wie dies in den Studien LoVAS, ADVOCATE und PEXIVAS erfolgte [32–34]. Bei der LoVAS-Studie wurden MPO-ANCA-Patient*innen mit gut erhaltener Nierenfunktion (52,0 gegenüber 55,3 ml/min/1,73 m^2^) per Randomisierung einer Standardgruppe und einer Gruppe mit reduzierter GC-Gabe zusätzlich zu RTX zugewiesen. Dabei stellten die Autoren ähnliche Remissionsraten über ein Follow-up von 6 Monaten fest, und die kumulative GC-Belastung betrug 1,32 gegenüber 4,15 g [32]. Das Ausschleichungsschema für GC ist in Tab. 4 zusammengefasst.

**Table 3:** 

	Orale CYC-Dosis (mg/kg/Tag)	Gepulste i.v. CYC-Dosis (mg/kg)^a^	
*eGFR* (ml/min/1,73 m^2^)	–	> 30	< 30
*Alter (Jahre)*			
< 60	2	15	12,5
60–75	1,5	12,5	10
> 75	1	10	7,5
*Protokoll*^b^	Tägliche Dosierung über 3 Monate; bis zu 6 Monate bei ausbleibender Remission	Gepulste Verabreichung in den Wochen 0, 2, 4, 7, 10, 13 und weitere gepulste Verabreichungen bei ausbleibender Remission	
*Maximale Dosis*	200 mg/Tag	1,2 g/gepulste Verabreichung	
*Großes Blutbild*	1. Monat: wöchentlich, 2–3 Monate: alle zwei Wochen, danach monatlich	Am Vortag oder am Tag der gepulsten Verabreichung	
*Therapieänderung*	In folgenden Fällen einstellen: – WBC < 3 oder Neutrophilen < 1,5 – Thrombozyten < 150	Wenn WBC **<** **4000/µl**, abwarten bis **>** **4000/µl** und die Dosis um 25 % reduzieren 10 bis 14 Tage nach gepulster Verabreichung FBC durchführen: Bei einem Nadir von < 3000/µl (auch wenn der Wert am Tag der gepulsten Verabreichung > 4000/µl ist) die Dosis wie folgt anpassen: – Wenn WBC 1000 bis 2000/µl, die Dosis um 40 % reduzieren – Wenn WBC 2000 bis 3000/µl, die Dosis um 20 % reduzieren	

*CYC* Cyclophosphamid, *eGFR* geschätzte glomeruläre Filtrationsrate, *FBC* großes Blutbild, *WBC* Leukozyten

^a^Gepulste Therapien werden aufgrund der geringeren kumulativen CYC-Dosis bevorzugt

^b^Angaben zur Dosierung von Prednisolon siehe Tab. 4

**Table 4:** 

	PEXIVAS (50–75 kg)			LoVAS	
Schema	Standard	Reduzierte Dosis		Hohe Dosis	Reduzierte Dosis
Pulstherapie	Ja	Ja	Pulstherapie	Nein	Nein
1. Woche	60 mg	60 mg	1./2. Woche	1,0 mg/kg	0,5 mg/kg
2. Woche	60 mg	30 mg	3./4. Woche	0,8 mg/kg	0,25 mg/kg
3./4. Woche	50 mg	25 mg	5./6. Woche	0,7 mg/kg	7,5 mg
5./6. Woche	40 mg	20 mg	7./8. Woche	0,5 mg/kg	5 mg
7./8. Woche	30 mg	15 mg	9./10. Woche	0,4 mg/kg	4 mg
9./10. Woche	25 mg	12,5 mg	11./12. Woche	0,35 mg/kg	3 mg
11./12. Woche	20 mg	10 mg	13.–16. Woche	15 mg	2 mg
13./14. Woche	15 mg	7,5 mg	17.–20. Woche	12,5 mg	1 mg
15./16. Woche	10 mg	5 mg	21.–24. Woche	10 mg	0 mg
17./18. Woche	10 mg	5 mg			
19./20. Woche	7,5 mg	5 mg			
21./22. Woche	7,5 mg	5 mg			
23.–52. Woche	5 mg	5 mg			
> 52. Wochen	Versorgung nach der jeweiligen Praxis des Prüfarztes				

Die PEXIVAS-Studie lieferte Hinweise darauf, dass ein zusätzlicher Plasmaaustausch (PLEX) neben der Standardtherapie keinen Einfluss auf die Sterblichkeit sowie Frequenz der Dialysepflicht der Patient*innen hat [34]. Teilanalysen ergaben jedoch, dass Patient*innen mit einem Serumkreatininwert > 5,7 mg/dl (> 500 µmol/l) eine signifikant reduzierte Quote an terminaler Niereninsuffizienz (ESKD) aufweisen, wobei die behandlungspflichtige Zahl (NNT) bei etwa 6 liegt, um einen ESKD-Fall im ersten Jahr zu reduzieren [35]. Dies unterstreicht die Ergebnisse der MEPEX-Studie, die ebenfalls einen kurzfristigen Nutzen hinsichtlich des Nierenüberlebens bei Patient*innen aufzeigte, bei denen PLEX durchgeführt wurde. Da bei Patient*innen, die eine PLEX-Therapie neben anderen immunsuppressiven Maßnahmen erhalten, vermehrt schwere Infektionen auftreten, sollten solche Patient*innen in entsprechend spezialisierten Zentren behandelt werden. Bei Patient*innen mit besonders schwerwiegendem Krankheitsbild (Serumkreatinin > 5,7 mg/dl, weitere Verschlechterung der Nierenfunktion trotz intensiver Immunsuppression) empfehlen wir die Durchführung von PLEX zusätzlich zu RTX oder CYC sowie die intravenöse Verabreichung von Methylprednisolon (3 Mal 500 mg), gefolgt von einem Ausschleichen der GC gemäß PEXIVAS-Studie (siehe Tab. 4). Eine Kombination von CYC (niedrige Dosis, 500 mg 6 Mal) und RTX (2 Mal 1000 mg) kann in solchen Szenarien in Betracht gezogen werden und würde eine noch schnellere Ausschleichung der oralen GC ermöglichen (GC-Absetzen nach 14 Tagen oder gemäß LoVAS) [36, 37] (Vergleiche siehe Tab. 4). Ein routinemäßiger PLEX zur Behandlung alveolärer Hämorrhagie bei GPA und MPA wird nicht empfohlen. Eine Teiluntersuchung der PEXIVAS-Studie ergab jedoch eine Reduktion der Mortalität bei Patient*innen mit schwerer alveolärer Hämorrhagie, bei denen ein PLEX durchgeführt wurde [38]. Selbstverständlich ist die Intensität der Immunsuppression an die Gegebenheiten der jeweiligen Patient*innen anzupassen, insbesondere unter Berücksichtigung von Begleiterkrankungen bei Patient*innen, die zu Komplikationen wie Infektionen neigen (vorbestehende Lungenerkrankungen, DM, Zunahme von Infektionskrankheiten usw.).

Bei Patient*innen mit Nierenerkrankung und *erhaltener* Nierenfunktion (eGFR > 15 ml/min/1,73 m^2^) empfehlen wir die Anwendung von RTX als Induktionstherapie, insbesondere bei Patient*innen mit PR3-ANCA-Vaskulitis und einem rezidivierenden Krankheitsverlauf [39, 40]. In einer randomisierten kontrollierten Studie wurde Mycophenolat-Mofetil (MMF) mit CYC verglichen und eine Nichtunterlegenheit von MMF in Bezug auf die Remissionserzielung nach 6 Monaten wurde festgestellt, wobei die Häufigkeit einer Remission 67 % in der MMF- und 61 % in der CYC-Gruppe betrug. Hierbei traten Rezidive bei Patient*innen mit PR3-ANCA-Vaskulitis in der MMF-Gruppe häufiger auf [41]. Daher sollte MMF eher Patient*innen mit MPO-ANCA-Erkrankung vorbehalten sein, insbesondere solchen mit Nierenbeteiligung [42]. Patient*innen mit einem Kreatininwert unter 1,7 mg/dl (150 μmol/l) wurden im Rahmen einer Studie entweder einer Behandlung mit oralem CYC (2 mg/kg täglich, Zieldosis) oder oralem Methotrexat (MTX) in einer Dosis von 20–25 mg pro Woche zugewiesen. In beiden Behandlungsarmen wurden hohe Remissionsraten erzielt: 93,5 % in der CYC-Gruppe und 89,8 % in der MTX-Gruppe. Auch hier wurden bei Patient*innen, die MTX erhielten, signifikant höhere Rezidivraten während einer Nachbeobachtungszeit von 18 Monaten beobachtet [43]. An dieser Stelle sei anzumerken, dass weder MMF noch MTX im Vergleich zu RTX untersucht wurden und dass die Unterschiede bei den Remissionsraten auf unterschiedlichen Definitionen beruhen, die bei diesen Studien verwendet wurden. Das Anwendungsfenster für MTX ist kleiner, insbesondere bei schwerer Nierenerkrankung, denn es ist auf Patient*innen mit einer eGFR > 30 ml/min/1,73 m^2^ beschränkt [43, 44]. In unserer Praxis beschränken wir die Verwendung von MTX auf Patient*innen, bei denen keine anhaltende schwere Nephritis (aktives Urinsediment, einschließlich Erythrozytenzylinder) vorliegt. Darüber hinaus erhielten Patient*innen in der NORAM-Studie hohe kumulative GC-Dosen, und zwar 8,8 g über einen Zeitraum von 18 Monaten in der MTX-Gruppe, was nicht mehr der heutigen Praxis entspricht. Dieses Ergebnis stellt die Anwendung von MTX bei AAV-Patient*innen weiter in Frage.

Die langfristigen Remissionsraten für MMF oder MTX sind zwar möglicherweise geringer als für CYC oder RTX, jedoch könnte die kürzere Wirkweise dieser Substanzen in einer pandemischen Situation von Interesse sein, da RTX und CYC mit erhöhten Sterblichkeitsraten assoziiert sind [45] und die Wirksamkeit einer Impfung nach der Behandlung mit RTX deutlich herabgesetzt ist (allgemeine Empfehlungen siehe Abb. 1; [46]). Avacopan, ein Hemmer des Komplement-C5a-Rezeptors 1, ist eine Alternative zu GC bei Patient*innen mit einer eGFR von über 15 ml/min/1,73 m^2^, die eine Nichtunterlegenheit gegenüber einem 20-wöchigen GC-Zyklus nach 6 Monaten und eine Überlegenheit nach 12 Monaten zeigte. Dabei war der Anstieg der eGFR in der Avacopan-Gruppe stärker, ebenso wie die frühe Verringerung von Albuminurie [33, 47]. Aufgrund der um ein Vielfaches höheren Behandlungskosten gegenüber GC kann dies eine Option für bestimmte Patient*innen sein, zum Beispiel bei Patient*innen mit hohem Risiko für schwere GC-induzierte Nebenwirkungen oder eingeschränkter Nierenfunktion. Das Management der „begrenzten“ Erkrankung ist umstritten, und die Beseitigung eines Befalls mit *Staphylococcus aureus* (Mupirocin-Salbe) wird mitunter nahegelegt. Unsere Gruppe ist jedoch der Meinung, dass der Behandlungsansatz immunsuppressive Wirkstoffe wie MTX, Azathioprin oder MMF enthalten sollte. In den Empfehlungen der EULAR 2022 wird auch die Verwendung von RTX in einem solchen Szenario empfohlen [27], was wahrscheinlich die Notwendigkeit einer niedrigeren kumulativen GC-Belastung im Vergleich zu den zuvor genannten Wirkstoffen widerspiegelt, um eine Krankheitsaktivität einzustellen.

Bei älteren Patient*innen wurde ein signifikanter Überlebensnutzen bei denjenigen festgestellt, die eine Induktionstherapie erhielten [48]. Die Intensität der Therapie ist jedoch auf den Gesundheitszustand jeder Person anzupassen. In der CORTAGE-Studie wurden Patient*innen ab 65 Jahren untersucht, die niedrig dosiertes CYC (6 Mal 500 mg) und eine GC-Therapie über 9 Monate als experimentelle Therapie erhielten. Während die Remissionsraten mit denen des Studienarms der konventionellen Behandlung vergleichbar waren, konnte die Anzahl der Patient*innen, bei denen mindestens ein schweres unerwünschtes Ereignis auftrat, reduziert werden [49].

Die Randomisierung von Patient*innen hinsichtlich ihres Einschlusses in klinische Studien basiert bisher nicht auf dem Parameter der Nierenhistologie. Eine histologische Untersuchung zu Studienbeginn kann eine Vorhersage über eine mögliche Wiederherstellung der Nierenfunktion ermöglichen, liefert jedoch keine Anhaltspunkte zur Bestimmung des Risikos für einen refraktären Krankheitsverlauf. In Übereinstimmung mit dieser Annahme fanden frühere Studien heraus, das Patient*innen mit halbmondförmiger Glomerulonephritis häufiger eine Remission erreichten, obwohl diese Form der ANCA-Glomerulonephritis als die „aggressivste“ Form gilt [50]. Höhere Chronizität-Scores deuten jedoch unabhängig von der verwendeten Behandlung (RTX oder CYC) auf geringere Wiederherstellungsraten der Nierenfunktion sowie ein höheres Risiko für ESKD/Tod und ein geringeres Ansprechen auf die Behandlung hin [19].

## Rezidive

Bei der Mehrzahl der Patient*innen mit AAV kommt es nach einer wirksamen Induktionstherapie zu einer Remission. Jedoch kommt es bei einer erheblichen Anzahl (fast 50 %) [51] der Patient*innen innerhalb von fünf Jahren nach der Diagnose zu einem Rezidiv, und dies geht mit einer Zunahme der krankheits- und therapiebezogenen Morbidität und Mortalität einher [52]. Eine Subanalyse der RAVE-Studie zeigte, dass RTX bei Patient*innen mit Rezidiv, insbesondere bei PR3-ANCA-Vaskulitis, gegenüber CYC/AZA überlegen ist [40]. Hier sei anzumerken, dass nur eine Minderheit der Patient*innen mit rezidivierender Erkrankung, die in die RAVE-Studie aufgenommen wurden, MPO-ANCA-positiv waren. Deshalb fehlen in dieser Teilgruppe wichtige Informationen [39]. Für andere Therapien fehlen derzeit Wirksamkeitsbeurteilungen, und diese Therapien werden bei Patient*innen mit rezidivierender Erkrankung nicht empfohlen.

## Refraktäre Erkrankung

Eine Erkrankungs-Refraktärität ist definiert als das Nicht-Ansprechen auf die verordnete Immunsuppression. In einer solchen Situation ist die Überweisung an ein Fachzentrum mit entsprechenden Kompetenzen zur AAV-Behandlung absolut notwendig, wobei wir generell zur Behandlung in einem Fachzentrum raten. Eine kurzzeitige Steigerung der GC-Dosis (und schließlich die zusätzliche Gabe von Methylprednisolon) kann vor der Überweisung sinnvoll sein. Im Allgemeinen kann eine Kombination mehrerer Therapien (insbesondere CYC und RTX) oder Zugabe von Avacopan zu einer Reduzierung der Krankheitsaktivität führen. Spezifische Strategien wurden bereits im Abschnitt Induktionstherapie beschrieben. Bei therapierefraktären Erkrankungen sollten andere Erkrankungen durch sorgfältige Differentialdiagnose ausgeschlossen werden.

## Erhaltungstherapie der ANCA-assoziierten Vaskulitis

Die Bestimmung optimaler Strategien zur Erhaltungstherapie bei Remission und das Identifizieren von Patient*innen, denen eine längere Erhaltungstherapie zugutekommt, sind weiterhin Herausforderungen. In RCT wurden mehrere Therapieformen zur Remissionserhaltung untersucht, darunter RTX, Azathioprin (AZA), MTX, MMF oder Belimumab. Generell sollte die Entscheidung über die optimale Erhaltungstherapie auf individuellen patientenbezogenen Faktoren und Präferenzen beruhen, wie z. B. vorangegangene Therapien, allgemeines Rezidivrisiko, Alter, Begleiterkrankungen oder Zugang zur Therapie.

Patient*innen, die entweder RTX oder CYC als Induktionstherapie erhielten, sollten für eine RTX-Erhaltungstherapie in Betracht gezogen werden. In der MAINRITSAN-Studie wurde nach einer Remissionsinduktion mit CYC eine feste Dosis von 500 mg RTX (an den Tagen 0 und 14 und in den Monaten 6, 12 und 18 nach Randomisierung) oder AZA (2 mg/kg/Tag über 12 Monate, gefolgt von mehreren Dosisverringerungen) verabreicht. Der Patientenanteil mit schweren Rezidiven war in der RTX-Gruppe deutlich geringer als in der AZA-Gruppe (5 % vs. 29 %) [53]. Bei der RITAZAREM-Studie wurden AAV-Patient*innen mit Rezidiv nach RTX-Induktionstherapie über 4 Monate mit 1000 mg RTX im Vergleich zur täglichen AZA-Gabe (2 mg/kg/Tag) behandelt, und auch hier wurde in der RTX-Gruppe eine geringere Rate an schweren Rezidiven festgestellt (13 % vs. 33) [54]. Wichtig ist, dass eine längere Anwendung von RTX über 4 Jahre zu einer anhaltenden Remission führen kann [55] und bei Patient*innen mit hohem Rezidivrisiko, wie z. B. bei Patient*innen mit PR3-AAV, Lungenbeteiligung oder HNO-Erkrankung, persistierendem oder wiederkehrendem ANCA-Titer oder häufig rezidivierender Erkrankung, in Betracht gezogen werden sollte. Die optimale Dauer und Dosierung einer verlängerten RTX-Erhaltungstherapie sind jedoch unklar. Aktuell sind die Bestimmung der zirkulierenden CD19+ B‑Zellen und der ANCA-Titer keine zuverlässigen Prädiktoren für eine kurzfristige Anpassung einer RTX-Erhaltungstherapie [56]. Ferner fehlt es an Langzeitdaten, und bei Patient*innen mit B‑Zellen-Depletion und fehlenden ANCA-Antikörpern kann in seltenen Fällen die Erkrankung rezidivieren [54, 57]. Aus diesem Grund empfehlen wir eine fest vorgegebene RTX-Dosierung anstelle einer Biomarker-gesteuerten, und die Dauer der Erhaltungstherapie sollte auf individueller Abwägung des Nutzens und der Risiken basieren. Die Biomarker-gesteuerte RTX-Behandlung sollte spezialisierten Zentren vorbehalten werden, die über Erfahrung mit dieser Behandlungsmethode verfügen. Dies wird auch in den EULAR-Empfehlungen 2022 erwähnt [27]. Die Erhaltungstherapie sollte über mindestens 24 Monate angewendet werden. Neuere Daten der Mayo Clinic zeigten jedoch, dass bei Patient*innen mit einem anhaltend negativen MPO-ANCA-Titer eine geringe Wahrscheinlichkeit für ein Rezidiv besteht und dass diese Patient*innen möglicherweise keine verlängerte Erhaltungstherapie benötigen [58]. Im Falle von Erkrankungen, die nicht auf gängige Immunsuppression ansprechen oder die mehrfach rezidivieren, ist die Überweisung an eine erfahrene Einrichtung ratsam.

Für AZA und MTX liegen mehr Daten zum Langzeitergebnis und zur Sicherheit vor. Wenn diese Substanzen angewendet werden, sollten individuelle Präferenzen in Betracht gezogen werden, um die Wahl zwischen diesen Wirkstoffen zu lenken. AZA und MTX zeigten eine ähnliche Wirksamkeit bei der Erhaltungstherapie bei AAV-Patient*innen, jedoch traten bei mit AZA behandelten Patient*innen tendenziell weniger unerwünschte Ereignisse auf (11 % vs. 19 %) [44]. Die Dauer einer Erhaltungstherapie mit AZA wurde in zwei RCT untersucht. In der AZA-ANCA-Studie war eine verlängerte Behandlung (2 vs. 4 Behandlungsjahre) mit einem längeren rezidivfreien Überleben verbunden. Dieser Zusammenhang war jedoch nicht signifikant [59]. Andererseits kam es in der REMAIN-Studie, an welcher mehr Patient*innen teilnahmen, bei den gleichen Behandlungszeiträumen in der Gruppe mit 2 Jahren Behandlung zu mehr Rezidiven. Allerdings wurde in der Gruppe mit der verlängerten Behandlung ein Sicherheitssignal identifiziert, das auf eine mögliche Häufung von Neutropenie und kardiovaskulären (CV) Ereignissen schließen lässt [60]. Die Erhaltungstherapie mit AZA könnte bei Patient*innen mit Kontraindikationen gegen RTX die bevorzugte Therapieoption bleiben. Insbesondere war MMF in der IMPROVE-Studie AZA unterlegen [61] und sollte nur angewendet werden, wenn Kontraindikationen gegen andere Wirkstoffe bestehen.

Besondere Aufmerksamkeit sollte älteren Patient*innen (> 75 Jahre) gelten, da bei diesen Patient*innen Rezidive weniger häufig aufzutreten scheinen, sie aber dennoch von einer Erhaltungstherapie profitieren könnten. Daten aus retrospektiven Analysen zeigten, dass die rezidivfreie Überlebenszeit bei Anwendung einer Kombination aus GC und Immunsuppressiva (im Vergleich zu GC allein) länger war [62], während eine reduzierte GC-Dosis mit weniger therapieinduzierten unerwünschten Ereignissen assoziiert ist [63]. Deshalb könnte bei dieser Patientenpopulation eine immunsuppressive Erhaltungstherapie mit einer reduzierten GC-Initialdosis in Kombination mit einem zusätzlichen immunsuppressiven Wirkstoff von Vorteil sein und sollte deshalb in Betracht gezogen werden.

## Follow-up

Die ersten Follow-up-Termine sollten auf die Bedürfnisse des Patienten zugeschnitten werden und hängen vom klinischen Schweregrad (d. h. Nierenfunktionsstörung) ab. Mit dem Patienten muss eine klare Strategie zum Ausschleichen der GC besprochen werden, und die Natur der Erkrankung, ihr natürlicher Verlauf sowie ihr Verlauf unter Immunsuppression/nach dem Absetzen der Behandlung sollten erörtert werden. In den ersten Jahren nach der Diagnose sollten Patient*innen Termine alle 3 Monate wahrnehmen. Diese Termine sollten eine klinische Untersuchung, Blutdruckmessung (oder Kontrolle des dokumentierten Blutdrucks), grundlegende Befragung zur Lebensqualität und eine umfangreiche Laboranalyse (einschließlich Entzündungsparameter, ANCA-Titer, Ig, Nierenfunktion usw.) vorsehen. Die meisten Krankheitsrezidive manifestieren sich in denselben Organsystemen, die bereits bei der Erstdiagnose betroffen waren.

## Therapiebedingte Komplikationen und Begleiterkrankungen bei ANCA-assoziierter Vaskulitis

### Infektionen

Infektionen sind nach wie vor ein ernstes Problem bei der Behandlung von Patient*innen mit AAV, die zu einer erhöhten Sterblichkeit beitragen [2, 64]. Eine Analyse der Risikofaktoren, die Infektionen vorhersagen, erfolgte hauptsächlich in Beobachtungsstudien, und es wurden verschiedene Risikofaktoren (Alter, Lungenbeteiligung, erhöhte Kreatininwerte bei Erstdiagnose, usw.) ermittelt [65, 66]. Neuere Studien deuten auf ein höheres Risiko für schwere Infektionen (SI) bei Patient*innen unter GC hin [67, 68]. Analog dazu zeigten Daten aus den Studien PEXIVAS und LoVAS eine deutliche Risikoverringerung für SI bei Patient*innen, die reduzierte GC-Dosen erhielten, ohne dass dies die Wirksamkeit herabsetzte [32, 34]. Bei der Mehrzahl der SI-Fälle handelte es sich um Infektionen der unteren Atemwege, und die Lungenbeteiligung war ein Prädiktor für Infektionen, während die Anwendung von Trimethoprim-Sulfamethoxazol (TMP/SMX) eine SI verhinderte (Verringerung um 70 %) [66]. Diese Daten unterstreichen die wichtige Rolle der TMP/SMX-Prophylaxe bei der Vorbeugung von infektiösen unerwünschten Ereignissen bei AAV, insbesondere frühzeitig nach der Behandlung mit RTX. Bisher existieren jedoch keine prospektiven Beobachtungsstudien oder kontrollierten Studien zur Dauer und Wirksamkeit von TMP/SMX, während in frühen Studien potenzielle Nebenwirkungen [69] und wirkstoffresistente Infektionen beobachtet wurden [70]. Insbesondere kann die Anwendung von hochdosiertem TMP/SMX (mit einer Dosis von 800/160 mg zweimal täglich) auch eine mögliche Wechselwirkung mit MTX hervorrufen, weshalb regelmäßige Blutbildanalysen erforderlich sind. In diesem Zusammenhang sei zu beachten, dass in anderen Studien weniger Nebenwirkungen beobachtet wurde, wenn eine verringerte prophylaktische TMP/SMX-Dosis verwendet wurde [71, 72]. Es sind kontrollierte Studien notwendig. Jedoch wird die prophylaktische Anwendung von TMP/SMX (d. h. 960 mg dreimal wöchentlich) während der Remissionsinduktionstherapie für alle Patient*innen mit hohem SI-Risiko empfohlen, z. B. bei Patient*innen mit Begleiterkrankungen, Lungenbeteiligung oder Behandlung mit hoch dosierten GC (> 15 mg Prednisolon oder gleichwertig über > 2 bis 4 Wochen) [73], CYC oder RTX. Bei Patient*innen unter RTX-Erhaltungstherapie sollte eine längere prophylaktische Anwendung in Betracht gezogen werden. Die Lymphozytenzahl im peripheren Blut und insbesondere die Bestimmung der CD4+-T-Zellen können in gewissen Fällen helfen, über die Notwendigkeit einer TMP/SMX-Behandlung als PJP-Prophylaxe zu entscheiden, wobei ein Wert von < 200 CD4+-T-Zellen für die Notwendigkeit einer PJP-Prophylaxe spricht [74]. Eine Reduzierung der TMP/SMX-Dosis ist bei Patient*innen mit schwerer Nierenerkrankung und einer eGFR < 30 ml/min/1,73 m^2^ notwendig. Zur Verhinderung weiterer infektiöser Komplikationen sollten Impfungen verabreicht werden (siehe nachstehende Erläuterungen). Bei Patient*innen, die während der Remissionsinduktion oder während einer RTX-Erhaltungstherapie positiv auf SARS-CoV‑2 getestet wurden und keinen nennenswerten humoralen Schutz aufweisen, sollte eine frühzeitige Behandlung mit monoklonalen Antikörpern und/oder einer antiviralen Therapie in Betracht gezogen werden. Bei Patient*innen mit zurückliegender HBV-Infektion besteht das Risiko einer Reaktivierung, wenn eine Anti-CD20-Therapie eingeleitet wird. Sie sollten durch entsprechende Leberfunktionstests und/oder Bestimmung der HBV-Viruslast überwacht werden [73]. Ferner wird das Management weiterer Risikofaktoren für Infektionen wie Hypogammaglobulinämie oder spätmanifeste Neutropenie (LON) nachstehend erörtert.

## Hypogammaglobulinämie

Eine Hypogammaglobulinämie tritt häufig bei Patient*innen mit AAV auf, besonders häufig bei RTX-Behandlung, und kann zu infektiösen Komplikationen führen. Die Daten zu den diesbezüglichen Risikofaktoren sind widersprüchlich. Generell besteht bei Patient*innen mit niedrigeren IgG Serumkonzentrationen bei der Erstdiagnose möglicherweise ein höheres Risiko für die Entwicklung einer schweren Hypogammaglobulinämie unter RTX-Therapie [75–77]. Zusätzlich ist bei der Langzeitbeobachtung eine frühere CYC-Behandlung oder längere GC-Einnahme mit der Entwicklung einer Hypogammaglobulinämie assoziiert [78]. Während die kumulative CYC-Dosis mit einer höheren Wahrscheinlichkeit für Hypogammaglobulinämie assoziiert sein könnte, wurde für RTX keine dosisabhängige Risikozunahme nachgewiesen [75, 76, 79, 80]. In der RITAZAREM-Studie trat bei Patient*innen, die AZA als Erhaltungswirkstoff erhielten, und solchen, die RTX nach einer RTX-Induktion erhielten, Hypogammaglobulinämie in ähnlicher Häufigkeit auf [54]. Eine Hypogammaglobulinämie ist in der Regel harmlos und vorübergehend, kann in seltenen Fällen jedoch auch schwerwiegend und möglicherweise mit SI assoziiert sein [75, 81]. Vor diesem Hintergrund kann bei Patient*innen ein IgG-Ersatz möglicherweise sinnvoll sein [82]. Bisher wurde jedoch in keiner kontrollierten Studie der Nutzen einer intravenösen (oder subkutanen) Gabe von Ig (IVIG) bei AAV-Patient*innen mit Hypogammaglobulinämie untersucht.

Wir empfehlen eine Messung der IgG-Serumspiegel bei allen Patient*innen vor der Induktionstherapie und während des Follow-ups. In Übereinstimmung mit anderen europäischen Konsensleitlinien [83, 84] könnte bei Patient*innen mit Hypogammaglobulinämie auf der Grundlage vorgeschlagener Empfehlungen zur Behandlung einer sekundären Hypogammaglobulinämie ein intravenöser oder subkutaner Ersatz von Immunglobulinen empfohlen werden [85].

## Spätmanifeste Neutropenie und Lymphopenie

Knochenmarksuppression ist eine bekannte Komplikation unter immunsuppressiven Therapien, die routinemäßig bei AAV angewendet werden. Eine spätmanifeste Neutropenie (LON) kann zu jedem Zeitpunkt während oder nach einer Phase der B‑Zell-Depletion auftreten und stellt eine schwere Nebenwirkung dar. In einer retrospektiven Analyse von 59 AAV-Patient*innen, die RTX erhielten, entwickelten 7 (11,9 %) eine LON (mittlere Zeit von 86 Tagen nach Beginn der RTX-Behandlung), was in fast allen Fällen zu einer Krankenhauseinweisung führte [86]. Andere Daten deuten allerdings darauf hin, dass die meisten Fälle selbstbegrenzend und von kurzer Dauer, sind [87]. Das Auftreten einer LON kann idiosynkratisch sein, und die prädisponierenden Faktoren sind unklar. In schweren Fällen ohne oder mit Fieber erwies sich die Gabe von rekombinantem Granulozyten-Kolonie-stimulierendem Faktor als wirksam [87]. Obwohl nur begrenzte Daten vorliegen, wird ein Absetzen von RTX im Fall von LON meist nicht empfohlen; es ist jedoch zu beachten, dass ein Rezidiv bei etwa 20 % der Patient*innen berichtet wurde [87].

Es muss ferner beachtet werden, dass eine verminderte Lymphozytenzahl vor Behandlung (< 800 mm^3^) und eine absolute Lymphopenie, die durch die zugrunde liegende Krankheit hervorgerufen wurde oder während der immunsuppressiven Therapie auftritt, mit schweren infektiösen Komplikationen assoziiert sind [88, 89]. In einer retrospektiven Studie war die CD4-Zahl in peripherem Blut ein unabhängiger Risikofaktor für Infektionen, mit einem besseren prädiktiven Wert für infektiöse Komplikationen als die Gesamtzahl an Lymphozyten (TLC) [90]. Aus diesem Grund sollte eine regelmäßige Kontrolle der TLC- und peripherer CD4-Werte während einer immunsuppressiven Behandlung erfolgen. Im Falle einer schweren Lymphopenie oder einer anhaltenden Reduktion des peripheren CD4-Wertes unter 200 Zellen/µl sollte eine prophylaktische antiinfektiöse Behandlung zur Vorbeugung opportunistischer Infektionen eingeleitet werden.

## Fruchtbarkeit

Die Behandlung mit GC kann die Ovarfunktion beeinflussen und bei Frauen zu Menstruationsstörungen und Unfruchtbarkeit führen [91], während die Daten zur männlichen Fruchtbarkeit widersprüchlich sind [92]. Bisher ist für RTX keine Auswirkung auf die Fruchtbarkeit bekannt, weder bei Frauen noch bei Männern. CYC kann hingegen bei beiden Geschlechtern solche Komplikationen zur Folge haben. Es wird über verschiedene Häufigkeiten berichtet, die von 5 bis nahezu 80 % reichen und vom Patientenalter und der kumulativen CYC-Dosis abhängen, wobei letztere der wichtigste Risikofaktor für CYC-induzierte Gonadentoxizität ist [93, 94]. Eine Behandlung mit Gonadotropin-Releasing-Hormon-Agonisten könnte bei Frauen im gebärfähigen Alter, die CYC erhalten, das Risiko einer Gonadentoxizität senken [95]. Für Patient*innen mit AAV liegen jedoch nur begrenzte Daten vor. Bei Männern kann die Reproduktionsfähigkeit durch Spermienkonservierung erhalten werden. Patient*innen, die eine zukünftige Schwangerschaft planen, wird die Beratung durch einen Reproduktions-Endokrinologen und/oder Fruchtbarkeitsspezialisten bezüglich der verfügbaren Behandlungsoptionen zum Erhalt der Fruchtbarkeit empfohlen. Da CYC, MMF und MTX teratogen sind, wird eine konsequente Empfängnisverhütung während dieser Therapien und mindestens 3 Monate darüber hinaus (oder ggf. gemäß den lokalen oder Herstellerempfehlungen) dringend empfohlen. Patient*innen die während dieser Behandlungen ungeplant schwanger werden, sollten zur weiteren Versorgung an spezialisierte Geburtshilfezentren überwiesen werden. Die Einnahme von GC und AZA zum Zeitpunkt der Empfängnis und während der Schwangerschaft gilt als sicher oder ist mit geringem Risiko behaftet, während RTX aufgrund seiner Auswirkungen auf das fetale Immunsystem und der ungenügenden Evidenzlage vor allem im zweiten und dritten Schwangerschaftsdrittel vermieden werden sollte [96]. Ein kürzlich veröffentlichter Konsensbericht über Immunsuppressiva und Biologika während Schwangerschaft und Stillzeit von den Österreichischen Gesellschaften für Gastroenterologie und Hepatologie sowie Rheumatologie und Rehabilitation ist verfügbar [97].

## Malignität

AAV sind mit einer Zunahme des allgemeinen Risikos für maligne Erkrankungen im Vergleich zur Allgemeinbevölkerung verbunden [98–101]. Dieses erhöhte Risiko könnte größtenteils mit dem karzinogenen Potenzial von immunsuppressiven Therapien zusammenhängen, insbesondere bei Patient*innen, die einer kumulativen Dosis von CYC > 36 g ausgesetzt waren (führt zu spätmanifestem Blasenkrebs, Leukämie oder „weißem“ Hautkrebs [NMSC]) [102, 103]. Hinsichtlich allgemeiner Malignität deuten mehrere jüngere epidemiologische Studien darauf hin, dass das Risiko mit dem der Allgemeinbevölkerung vergleichbar ist, was einen pathophysiologischen Zusammenhang oder übereinstimmende Verläufe zwischen AAV und malignen Erkrankungen vorwiegend ausschließt [104, 105]. Die beobachteten geringeren Krebsrisiken lassen sich durch die zunehmende Anwendung von RTX und die reduzierte Verabreichung von CYC erklären [102]. Dennoch bleibt das NMSC-Risiko erhöht, und wir empfehlen Patient*innen eine jährliche dermatologische Kontrolle. Zusätzlich wird empfohlen, Patient*innen aufzuklären, übermäßige Bestrahlung mit Ultraviolettlicht zu vermeiden, hochwirksamen Sonnenschutz zu verwenden und regelmäßige Selbstkontrollen auf Hautläsionen durchzuführen. Zusätzlich sollte bei Patient*innen mit früherer CYC-Behandlung eine regelmäßige Kontrolle auf Hämaturie (alle 3 bis 6 Monate) durchgeführt werden, um einen möglichen Blasenkrebs zu erkennen. Bei Patient*innen, die eine Langzeitbehandlung mit AZA erhalten, sollte auf ein erhöhtes Lymphomrisiko geachtet werden.

## Begleiterkrankungen bei ANCA-assoziierter Vaskulitis

Fortschritte in der Therapie führten zu einer deutlichen Verbesserung des Überlebens bei AAV-Patient*innen. Dennoch sind die Mortalitätsraten nach wie vor höher als in der Allgemeinbevölkerung, was zum Teil auf früh- und spätmanifeste Begleiterkrankungen zurückzuführen ist [106]. In der akuten Phase der Erkrankung ist das Risiko für venöse Thromboembolie-Ereignisse (VTE) erhöht (vor allem für tiefe Venenthrombose), und vor kurzem wurde über VTE-Häufigkeiten von bis zu 17 % berichtet [107, 108], insbesondere aufgrund von Hyperkoagulabilität, die auch in der Remission bestehen bleiben könnte [109]. Risikofaktoren für VTE sind unklar, dennoch sind Patient*innen mit positiven PR3-ANCA, Lungenblutungen sowie mit Beteiligung bestimmter Organe (d. h. Lunge, Niere, Herz, Haut) einem höheren Risiko ausgesetzt [107, 110, 111]. Daher könnte eine maßgeschneiderte Antikoagulationsprophylaxe nach Risiko-Nutzen-Stratifizierung (d. h. unter Berücksichtigung des Zeitpunkts der Therapieeinleitung oder des hohen Blutungsrisikos) auf individueller Basis für bestimmte AAV-Patient*innen nützlich sein.

Die Beteiligung des Herz‑/Kreislaufsystems gilt als kritischer Prognosefaktor, da diese Manifestation mit einem erhöhten Risiko für Herz‑/Kreislauf-Erkrankungen und höheren Mortalitätsraten einhergeht [112]. Insbesondere GC-Therapie, Komplikationen einer Atherosklerose, das Vorliegen einer chronischen Nierenerkrankung (CKD) und ein chronisch-entzündlicher Zustand könnten für das erhöhte kardiovaskuläre Risiko verantwortlich sein. Zusätzlich entwickelt ein hoher Anteil der AAV-Patient*innen einen DM, entweder als Folge der GC-Gabe oder in Verbindung mit einer zugrunde liegenden Erkrankung. Langzeit-Beobachtungsdaten aus Studien der Europäischen Vaskulitis-Gesellschaft zeigten, dass die Häufigkeit von DM von 1,1 % bei Erkrankungsbeginn auf 10,4 % nach 7,3 Jahren Nachbeobachtung anstieg [113]. Da DM einer der wichtigsten modifizierbaren kardiovaskulären Risikofaktoren ist, sind eine frühzeitige Erkennung und rechtzeitige Behandlung von entscheidender Bedeutung. Darüber hinaus ist ein weiteres multifaktorielles Management kardiovaskulärer Risikofaktoren, einschließlich Kontrolle von Dyslipidämie und Hypertonie, besonders bei Patient*innen mit Prädiabetes oder diagnostiziertem DM von wesentlicher Bedeutung. Daten der RAVE-Studie zeigten einen signifikanten Anstieg der Lipidspiegel (d. h. Gesamtcholesterin, LDL-Cholesterin und Apolipoprotein B) 6 Monate nach Behandlungsbeginn bei neu diagnostizierten PR3-AAV-Patient*innen [114]. Entsprechend waren Dyslipidämie und Hypertonie bei einer retrospektiven Kohorte von AAV-Patient*innen mit einem höheren Risiko schwerer kardiovaskulärer Ereignisse verbunden [115]. Während zunehmende Evidenz aufzeigt, wie wichtig eine Reduzierung des kardiovaskulären Risikos bei AAV ist, bleibt das Management der damit assoziierten Risikofaktoren ungenügend [116]. Bisher liegen keine prospektiven Studien oder kontrollierten Studien über das Management von Begleiterkrankungen bei AAV vor. Dennoch werden eine regelmäßige Kontrolle der herkömmlichen Risikofaktoren nach Framingham, Raucherentwöhnung, regelmäßige körperlicher Betätigung sowie Zielwerte für Blutdruck und Serumlipidspiegel gemäß den aktuellen KDIGO- und ESC-Leitlinien empfohlen [117, 118]. In ähnlicher Weise ist eine optimale glykämische Kontrolle unerlässlich, und Antidiabetika mit positiver Wirkung auf das Herz‑/Kreislaufsystem und den Nieren, wie etwa Glucagon-like-Peptid-(GLP)-1-Agonisten und Natrium-Glucose-Cotransporter-2-(SGLT-2)-Hemmern sollte der Vorzug gegeben werden [119].

Es gibt weitere, gut bekannte Risikofaktoren, die mit einer verlängerten GC-Anwendung bei AAV-Patient*innen verbunden sind, darunter negative Auswirkungen auf die Knochengesundheit. GC erhöhen das Frakturrisiko insbesondere aufgrund eines beschleunigten Knochenschwundes, der im ersten Jahr der Anwendung besonders ausgeprägt ist [120]. Bei AAV-Patient*innen wurde ein höheres Osteoporoserisiko beobachtet [121, 122], das jedoch auch eine Spätfolge der AAV-Behandlung und einer CKD sein kann [113, 123]. Für alle Patient*innen, die längerfristig mit oralen GC behandelt werden, wird eine Bestimmung des Frakturrisikos (d. h. Bestimmung der Knochenmineraldichte, Anwendung des FRAX-Risikoscores) empfohlen. Darüber hinaus sollte bei Patient*innen mit erhöhtem Risiko auf eine pharmakologische und nicht-pharmakologische Risikoprävention (d. h. Vitamin-D- und Calciumergänzung, Sturzprävention und Änderung der Lebensweise) geachtet werden, während bei Patient*innen mit stärkerem Frakturrisiko ein erweitertes pharmakologisches (z. B. Bisphosphonate) Management erforderlich ist.

In den vergangenen Jahren wurde das Auftreten einer interstitiellen Lungenerkrankung (ILD) bei AAV zunehmend erkannt. MPO-ANCA-positive Patient*innen scheinen ein höheres Risiko für das Auftreten einer ILD zu haben [124]. Wenn bei der hochauflösenden CT (HRCT)-Untersuchung des Thorax Anzeichen interstitieller Anomalien unbekannter Ätiologie festgestellt werden, empfiehlt sich eine multidisziplinäre Diskussion mit Radiologen und Pulmologen. Die Empfehlungen für das Screening und die Behandlung von Begleiterkrankungen bei AAV sind in der Tab. 5 zusammengefasst.

**Table 5:** 

Schwerpunkt	Beschreibung	Empfehlungen	
Infektionen	Personen mit AAV haben ein höheres Infektionsrisiko bei Einleitung einer systemischen Therapie. Infektionen der unteren Atemwege und der Harnwege sind häufige infektiöse Komplikationen. LON und Hypogammaglobinämie können auftreten, insbesondere bei Patient*innen, die mit CYC und RTX behandelt werden	1.	Das Infektionsrisiko sollte vor Beginn einer systemischen Therapie beurteilt werden (einschließlich des Phänotyps solcher Erkrankungen)
		2.	Alle Patient*innen sollten vor Beginn einer immunsuppressiven Therapie auf HBV und HCV untersucht werden
		3.	Eine prophylaktische Verwendung von TMP/SMX (d. h. 960 mg drei Mal pro Woche) wird bei Patient*innen mit einem hohen Risiko für schwere Infektionen empfohlen. Die Therapie sollte nach Therapiebeginn mindestens 3 Monate lang fortgesetzt werden. Bei Patient*innen, die eine RTX-Erhaltungstherapie und/oder eine tägliche Steroiddosis ≥ 15 mg über eine Dauer von > 2 bis 4 Wochen erhalten, sollte eine verlängerte prophylaktische Anwendung in Betracht gezogen werden. Mögliche Nebenwirkungen, die mit dieser Therapie verbunden sind, sollten überwacht werden
		4.	Die Anwendung von GC sollte minimiert werden, um schwere infektiöse Komplikationen zu vermeiden
		5.	Eine Impfung sollte nach Berücksichtigung möglicher Kontraindikationen durchgeführt werden, vor allem die jährliche Grippeimpfung und Impfung gegen Pneumokokken gemäß nationalen Empfehlungen. Die Häufigkeit der COVID-19-Impfung muss bestimmt werden
		6.	Eine IVIG-Substitution kann bei Patient*innen mit Hypogammaglobinämie, bei denen atypische oder rezidivierende schwere Infektionen auftreten, in Betracht gezogen werden
		7.	Eine IVIG-Substitution sollte unter Beachtung von relevanten Empfehlungen in Betracht gezogen werden
VTE	In der akuten Phase der Erkrankung ist das Risiko für VTE erhöht (hauptsächlich für eine tiefe Venenthrombose), welches auch während der Remission bestehen bleiben kann	1.	Das Risiko für VTE sollte vor Beginn einer systemischen Therapie beurteilt werden (einschließlich des Phänotyps solcher Erkrankungen), und eine VTE-Prophylaxe sollte von Fall zu Fall in Erwägung gezogen werden
		2.	Bei Patient*innen mit hohem Blutungsrisiko ist eine Antikoagulation zu vermeiden
Kardiovaskuläre Risiken	Personen mit AAV haben ein höheres Risiko für Herz‑/Kreislauferkrankungen als die Allgemeinbevölkerung. Die Krankheit selbst, unterschiedliche Therapieformen und klassische Risikofaktoren tragen zu diesem Risiko bei	1.	Ein Screening auf Hyperlipidämie wird empfohlen; die üblichen Leitlinien zur Prävention und Behandlung sollten befolgt werden
		2.	Ein Screening auf arterielle Hypertonie wird empfohlen; die üblichen Leitlinienlinien zur Prävention und Behandlung sollten befolgt werden
		3.	Ein Screening auf Prädiabetes und manifesten DM wird empfohlen; die üblichen Leitlinien zur Prävention und Behandlung sollten befolgt werden
		4.	Eine Raucherentwöhnung und regelmäßige körperliche Betätigung sollten empfohlen werden
		5.	Die Anwendung von GC sollte bei Patient*innen mit kardiovaskulären Risikofaktoren auf ein Minimum beschränkt werden
Malignität	AAV sind mit einer Zunahme des allgemeinen Risikos für maligne Erkrankungen im Vergleich zur Allgemeinbevölkerung verbunden. Das NMSC-Risiko bleibt unter allen gängigen immunsuppressiven Therapien erhöht. Bei Patient*innen, die mit CYC behandelt werden, stellt spätmanifester Blasenkrebs ein erhebliches Risiko dar	1.	Es wird empfohlen, Patient*innen aufzuklären, übermäßige Bestrahlung mit UV-Licht zu vermeiden, hochwirksamen Sonnenschutz zu verwenden und regelmäßige Selbstkontrollen auf Hautläsionen durchzuführen
		2.	Personen mit erhöhtem Hautkrebsrisiko benötigen möglicherweise eine engmaschigere Überwachung (d. h. jährliche dermatologische Kontrolle)
		3.	Bei Personen mit früherer CYC-Behandlung sollte eine regelmäßige Kontrolle auf Hämaturie durchgeführt werden, um Blasenkrebs zu erkennen
Osteoporose	Personen mit AAV haben ein höheres Osteoporose-Risiko als die Allgemeinbevölkerung, insbesondere Patient*innen, die GC erhalten	1.	Allen Patient*innen, die eine GC-Behandlung beginnen oder langfristig erhalten, wird eine Beurteilung des Frakturrisikos mit Messung der BMD und Verwendung des FRAX-Risikorechners empfohlen
		2.	Alle Patient*innen, die eine GC-Behandlung beginnen oder langfristig erhalten, sollten kontrolliert und über Strategien zur Verbesserung der Ernährung (d. h. Vitamin-D- und Kalziumaufnahme), zur Verringerung des Sturzrisikos und zu anderen Änderungen der Lebensweise beraten werden. Bezüglich der Prävention sind die üblichen Leitlinien zu befolgen
		3.	Alle Patient*innen mit erhöhtem Frakturrisiko sollten eine geeignete pharmakologische Behandlung erhalten. Bezüglich der Behandlung sind die üblichen Leitlinien zu befolgen
		4.	Zur Vermeidung von Knochenschwund sollte die Anwendung von GC auf ein Minimum reduziert werden
Interstitielle Lungenerkrankung	Bei Personen mit AAV besteht mitunter ein erhöhtes ILD-Risiko	1.	Spirometrische und DLCO-Messungen sollten bei allen Patient*innen durchgeführt werden
		2.	Bei Patient*innen mit auffälligen Lungenfunktionswerten sollte eine HRCT durchgeführt werden
		3.	Wenn bei der HRCT-Untersuchung Anzeichen interstitieller Anomalien unbekannter Ätiologie festgestellt werden, empfiehlt sich eine multidisziplinäre Diskussion zwischen Radiologen und Pulmologen
Fruchtbarkeit	Die Gonadentoxizität und Teratogenität, die mit bestimmten immunsuppressiven Therapien assoziiert sind, sind gut bekannt	1.	Patient*innen, die eine zukünftige Empfängnis planen, wird die Beratung durch einen Reproduktions-Endokrinologen und/oder Fruchtbarkeitsspezialisten bezüglich der verfügbaren Behandlungsoptionen zur Erhaltung der Fruchtbarkeit empfohlen
		2.	Eine konsequente Empfängnisverhütung während der Therapie mit CYC, MMF und MTX sowie mit anderen Wirkstoffen (wie ACEi) und mindestens 3 Monate darüber hinaus (oder ggf. gemäß den lokalen oder Herstellerempfehlungen) wird dringend empfohlen. Sollte es im Verlauf dieser Therapien zu einer ungeplanten Schwangerschaft kommen, sollte eine Überweisung zur weiteren Versorgung an ein spezialisiertes Geburtshilfezentrum erfolgen
		3.	GC und AZA gelten während der Schwangerschaft als sicher und können gegebenenfalls angewendet werden. Die Gabe von RTX während der Schwangerschaft sollte vermieden werden
		4.	Die nationalen und internationalen Empfehlungen zu Immunsuppressiva und Biologika während der Schwangerschaft und Stillzeit sollten befolgt werden

*AAV* ANCA-assoziierte Vaskulitiden, *ACEi* Angiotensin-Converting-Enzym-Hemmer, *AZA* Azathioprin, *BMD* Knochenmineraldichte, *CVD* kardiovaskuläre Erkrankung, *CYC* Cyclophosphamid, *DLCO* Diffusionskapazität von Kohlenmonoxid, *DM* Diabetes mellitus, *GC* Glucocorticoid, *HBV* Hepatitis-B-Virus, *HCV* Hepatitis-C-Virus, *HRCT* hochauflösende Computertomographie, *ILD* interstitielle Lungenerkrankung, *IS* Immunsuppression, *IVIG* intravenöse Immunoglobuline, *LON* late-onset Neutropenia (spätmanifeste Neutropenie), *MMF* Mycophenolat-Mofetil, *MTX* Methotrexat, *NMSC* nicht melanonzytärer Hautkrebs, *UV* Ultraviolett, *RTX* Rituximab, *TMP/SMX* Trimethoprim-Sulfamethoxazol, *VTE* venöse Thromboembolie

## Impfungen bei ANCA-assoziierter Vaskulitis

Wir empfehlen die Impfung von Patient*innen gemäß den aktuellsten nationalen Impfempfehlungen für Patient*innen mit Autoimmunerkrankungen: Hierzu gehören die jährliche Grippeimpfung, Pneumokokkenimpfung (Konjugatimpfstoff gefolgt von Polysaccharid-Impfstoff gegen Pneumokokken nach >/= 8 Wochen alle 3 bis 5 Jahre) [125] sowie COVID-19-Impfung. Wir empfehlen auch eine HBV-Impfung, idealerweise vor Beginn der RTX-Therapie, oder, wenn dies nicht möglich ist, nach einer Repopulation der B‑Zellen oder 4 Wochen vor dem anschließenden RTX-Zyklus. Im Zusammenhang mit der Impfung von AAV-Patient*innen ist es wichtig, einige spezifische Punkte zu beachten. Zunächst ist die Immunantwort auf die Impfung bei Patient*innen, die eine Induktionstherapie mit hohen GC-Dosen und eine zusätzliche Immunsuppression (wie CYC und RTX) erhalten, reduziert. In einer kleinen französischen Studie wurde festgestellt, dass nach 27 Monaten nur ein(e) (von elf) Patient*innen eine Immunität gegen Pneumokokken aufwies, wenn der Impfstoff während der Induktionstherapie verabreicht wurde, während die Immunität bei sieben von zehn Patient*innen intakt war, wenn der Impfstoff während der Erhaltungsphase, verabreicht wurde [126]. Die Einführung der COVID-19-Impfstoffe hat unser Verständnis der humoralen und zellulären Reaktion auf Impfstoffe bei AAV tiefgreifend verändert [46]. Risikofaktoren für eine mangelhafte humorale Reaktion sind Behandlungen mit RTX, MMF und höheren GC-Dosen (normalerweise ≥ 10 mg/Tag), während für andere Therapien, wie z. B. CYC, die Informationslage noch unzureichend ist und eine beeinträchtigte Impfreaktion möglicherweise zu erwarten ist. Es ist unklar, ob das Ausbleiben oder eine schwache Antikörperantwort einem fehlenden Schutz gegen COVID-19 gleichzusetzen ist; dies wurde insbesondere im Zusammenhang mit schweren Krankheitsverläufen bei ungeimpften Patient*innen beobachtet. Es gibt nach wie vor Diskussionen über die Auswirkungen der zellulären Reaktion, und die meisten Studien zeigten, dass eine zelluläre Immunantwort unter RTX-Therapie erhalten bleibt [127, 128]. Der ideale Zeitpunkt für eine Impfstoffverabreichung, eine flexible Verabreichung der Erhaltungstherapie und die gemeinsame Entscheidungsfindung zu Risiken und Nutzen eines Aussetzens der Therapie sind zur Realität geworden. Ferner muss beachtet werden, dass zwar über Einzelfälle neuer AAV-Erkrankungen nach der COVID-19-Impfung berichtet wurde [129], es jedoch keinen kausalen Zusammenhang zu geben scheint [130, 131]; und weitere Analysen sind im Gange, um aktuelle Daten zu den Inzidenzraten zu erhalten. Wir empfehlen, Patient*innen gegen COVID-19 zu impfen und im Falle einer ausbleibenden Impfantwort flexible Behandlungsansätze zu verwenden, wie z. B. die Verabreichung weiterer Booster-Dosen oder einen Wechsel von einem Vektorimpfstoff zu einem mRNA-Impfschema und umgekehrt.

## Fazit

In den letzten Jahrzehnten wurden die diagnostischen Möglichkeiten und das therapeutische Management von AAV verbessert. Nach Zulassung von RTX durch die Europäische Arzneimittel-Agentur wurde dieses Präparat vermehrt zur Remissionsinduktion angewendet; und seine zulassungskonforme Verwendung in der Erhaltungsphase hat zu einer deutlichen Verringerung der Rezidivrate von Patient*innen im Verlauf der Behandlungsperioden geführt. Mit dem Aufkommen alternativer Therapien, wie z. B. Avacopan, ist eine Verringerung oder Vermeidung der GC-Gabe in naher Zukunft möglicherweise denkbar, wodurch bei dieser Patientenpopulation zudem die negativen Wirkungen einer GC-Behandlung wie DM, Osteoporose oder Hypertonie verringert würden. Aktuelle Forschungsarbeiten beschäftigen sich mit der Optimierung der Langzeitprognose dieser Erkrankung. Hierzu gehören ähnliche Strategien, wie sie für das Management von CKD verwendet werden, z. B. eine SGLT-2-Hemmung und eine bessere Kontrolle von kardiovaskulären Risikofaktoren wie DM und Hypertonie. Insbesondere Avacopan hat in der ADVOCATE Studie zu einer deutlichen Verbesserung der Lebensqualität und einer verringerten GC-Toxizität geführt [33], ein weiterer wichtiger Schritt zur Verbesserung der körperlichen Leistungsfähigkeit von AAV-Patient*innen. Maßgeschneiderte Übungspläne und Physiotherapie zur Überwindung von Problemen (z. B. Gang- und Haltungsstörungen) sind hier ein logischer und notwendiger nächster Schritt, um die Therapie für unsere Patient*innen zu optimieren. Angesichts der rapiden Fortschritte der Forschung auf diesem Gebiet wird eine Aktualisierung der derzeitigen Empfehlungen in naher Zukunft notwendig sein.
